# Detection of DNA viruses in prostate cancer

**DOI:** 10.1038/srep25235

**Published:** 2016-04-28

**Authors:** Vitaly Smelov, Davit Bzhalava, Laila Sara Arroyo Mühr, Carina Eklund, Boris Komyakov, Andrey Gorelov, Joakim Dillner, Emilie Hultin

**Affiliations:** 1 International Agency for Research on Cancer, World Health Organization, Screening Group, Lyon, 69372, France; 2 Karolinska Institutet, Department of Laboratory Medicine, Stockholm, 141 86, Sweden; 3 North-Western State Medical University named after I.I. Mechnikov, Department of Urology and Andrology, St. Petersburg, 191015, Russia; 4 St. Petersburg State University, Faculty of Medicine, St. Petersburg, 199034, Russia

## Abstract

We tested prostatic secretions from men with and without prostate cancer (13 cases and 13 matched controls) or prostatitis (18 cases and 18 matched controls) with metagenomic sequencing. A large number (>200) of viral reads was only detected among four prostate cancer cases (1 patient each positive for Merkel cell polyomavirus, JC polyomavirus and Human Papillomavirus types 89 or 40, respectively). Lower numbers of reads from a large variety of viruses were detected in all patient groups. Our knowledge of the biology of the prostate may be furthered by the fact that DNA viruses are commonly shed from the prostate and can be readily detected by metagenomic sequencing of expressed prostate secretions.

Among risk factors proposed for prostate cancer^1^,^2^ are chronic inflammation of the prostate^3^,^4^,^5^ and sexually transmitted infections^6^,^7^,^8^,^9^,^10^,^11^. Expressed prostate secretions (EPS) are readily obtained and recommended samples for diagnosis of prostatic inflammation^12^,^13^. EPS are also informative for viral epidemiological studies^14^,^15^. Metagenomic sequencing (MGS) determines non-human sequences present in a sample without prior cloning and is fundamental for modern infectious disease epidemiology^16^,^17^,^18^,^19^. We previously reported that JC polyomavirus was readily detectable in EPS using MGS^20^. However, the MGS technology used at that time had a limited sequencing depth and some samples used also contained urine. To obtain a more complete picture of the viruses shed from the prostate, we wished to analyze pure EPS samples using modern MGS.

## Results

Of the total 927 million reads, 13974 reads (0.0014%) mapped to viruses. Odds ratios (ORs) and 95% confidence intervals (CIs), computed based on the Wald approximation, did not reveal any statistically significant differences between patient groups regarding presence of viral sequences (Tables 1 and 2).

Viral reads were detected in 7/13 cancer cases (2 to 7938 reads) and in 4/13 cancer controls (2 to 96 reads). There were 13654 viral reads in cancers, compared to only 170 reads in controls (Table 1). The study design considered individual patients and post hoc analyses of whether total number of reads differed between cases and controls were therefore not performed. There were only a few viral reads in 5/18 prostatitis cases (2 to 14 reads) and in 4/18 of their matched controls (4 to 46 reads) (Table 2).

A patient with 7938 reads of Merkel cell polyomavirus generated most of the viral reads, followed by a patient with 4478 reads of HPV type 89. The only other subjects with >200 viral reads were one cancer case with 286 reads of JC polyomavirus and a fourth cancer case with 222 reads of HPV type 40. JC polyomavirus was also detected in one additional cancer case (12 reads). Interestingly, the cancer controls had much less viral reads. One control was positive for both Epstein Barr virus (EBV) (88 reads) and JC polyomavirus (8 reads), another was positive for only EBV (14 reads) and a third was positive for HPV43 (40 reads).

## Discussion

We report that many prostate cancer patients shed virus DNA and that there were 4 subjects that had one clearly dominant viral species. Strengths of the study include novelty regarding use of modern, deep MGS with an average of 943 million reads per sample, at a 150 bp read length. This corresponds to a sequencing depth of the human genome of >40 times. The Illumina specification of the system promises only a 30 times sequencing depth of the huma genome, suggesting that a virus needs to be present in a proportion of about 1 virus copy per 30 human geneomes. Also, the fact that we studied a prostatic sample (EPS) that can be readily obtained also for large-scale, epidemiological studies as a strength.

Limitations include the limited number of observations. In particular, the higher number of viral reads that we found in cancer cases was based on a small number of subjects and may thus have been attributed to chance. The fact that MGS provides the exact sequence of the virus enables the study of viral subtypes in cases and controls, but the present study had too few positive observations for a meaningful analysis of subtypes. Also, the study was based on subjects that already have the disease. Thus, it is possible that presence of disease may cause presence of viruses rather than the opposite. For example, malignancy may have caused changes that are beneficial for viral replication. Also, since also the control subjects had PSA levels >4 ng/ml, there is a possibility that some of them are false-negative for prostate cancer even though the 18 core biopsy protocol used did not find any cancer^26^. Larger studies, preferably prospective studies, would be required to elucidate whether viral infections are involved in prostatic disease.

Most previous studies have only studied one or a few infections at a time and/or have used samples (e.g. prostatic biopsies) that are difficult to obtain on a large-scale and identical manner from cases and controls^6^,^8^,^9^,^10^,^11^,^27^. Although many studies have used MGS for comprehensive detection of viruses in human specimens such as skin samples^16^,^18^,^19^, serum^17^ or cervical cells^25^, as far as we know only a single previous study, from our lab, has used MGS on prostatic specimens^20^. That study was performed using a now outdated MGS technology^20^, but is consistent with our results for example regarding frequent detection of JC virus in EPS.

In summary, the knowledge that DNA viruses are commonly shed from the prostate and can be readily detected by MGS of EPS may further the possibilities to study the biology of the prostate.

## Materials and Methods

### Study design and patients

Thirteen men with prostate cancer were diagnosed among 100 visitors of an oncological dispensary. A standard TRUS-guided 18-core prostate biopsy with subsequent histopathological analysis was carried out in those with abnormal (>4.0 ng/ml) serum prostate-specific antigen (PSA). All cases included were newly diagnosed prostate cancer patients who had not received treatment and where diagnosis was based on the histopathological findings. Out of 100 men, 13 age-matched control patients histologically found to have non-malignant disorders of the prostate were included. The median age of the controls was 68 years (range 42–78), whereas the cases had a mean age of 70 years (range 55–79). EPS specimens were obtained 1–2 days before prostate biopsy. From 900 attendees of a urology unit at a genitourinary clinic, 18 patients with chronic (persisting for >3 months) inflammation of the prostate, without any microbiological agent detectable by standard methods, were age-matched with 18 healthy subjects from the same cohort^15^,^20^. The median age of the controls was 32 years, of the prostatitis cases 36 years. The prostatitis controls and cases were highly sexually active (average of 15–20 lifetime sexual partners), whereas the prostate cancer cases and controls all reported having had only 1 partmer for at least 10 years. Data on smoking, diet or exercising habits was not collected.

The institutional review boards, namely the Department of Clinical Investigations and Intellectual Property, of St. Petersburg Medical Academy of Postgraduate Studies (North-Western State Medical University named after I.I. Mechnikov since 2011) under the Federal Agency of Public Health and Social Development of Roszdrav approved the study. The methods were carried out in accordance with the approved guidelines. All participants provided written informed consent for this study.

### DNA isolation and Metagenomic sequencing

After the EPS samples had been frozen and thawed, DNA was extracted by boiling 5 uL of EPS sample diluted in 95 uL of 1 × TE-buffer at 107 °C for 10 min. All 62 samples together with 2 negative controls were subjected to random whole genome amplification using the Illustra Ready-2-Go GenomiPhi HY DNA Amplification kit (GE Healthcare, UK), following the manufacturer’s guidelines except that the incubation time was prolonged from the recommended 4 hours to 7 hours. Laboratory grade water (Sigma-Aldrich, St. Louis, MO, US) was used as negative control to assess false positive reads. After the amplification reaction, all samples was diluted 1:2 in water and quantified using QuantiFluor-ST (Promega, US)^20^, a fluorometric assay quantifying dsDNA, according to manufacturer’s user guide. DNA concentration after WGA ranged between 344 to 1288 ng/ul, with a median of 676 ng/ul. DNA libraries were prepared using the Nextera DNA Sample Preparation kit with the 96 index system according to the user guide revision B (Illumina), starting with 50 ng DNA in the tagmentation reaction. The library pools were quantified with the QuantiFluor system as above and the library sizes were checked using the Bioanalyzer High Sensitivity DNA chip (Agilent). DNA concentration after library prep ranged between 1.1 to 8.5 ng/ul, with a median of 3.3 ng/ul. Each library was normalized to 10 nM before pooling of all 64 libraries. The library pool was denatured and diluted to 2.6 pM and spiked with 1% PhiX control according to Denaturing and Diluting Libraries for the NextSeq 500 Rev. B manual (Illumina) prior to paired-end sequencing of 151+151 cycles (2 × 151 bp read length) on the NextSeq instrument and NCS v1.2 using NextSeq 500 High-Output Reagent Kit (Illumina), following NextSeq 500 System User Guide Rev. E (Illumina). The sequencing flow cell cluster density was 227 K/mm^2^ and the total yield was 147 Gb with 67% >Q30 and approximately 7.4 million paired-end reads passing filters per sample.

### Data analysis

Bioinformatic analyses used R (www.R-project.org) and python (www.python.org) scripts run on a 40 core, 2 TB RAM Linux server. Short index sequences (part of the Illumina primers) were used to assign sequences to the originating sample^21^. Sequences were quality checked and trimmed according to Phred quality scores^22^. Reads were screened against the human reference genome hg19 using BWA-MEM^23^ and reads with >95% identity over 75% of their length to human DNA were removed. Sequences were then normalized (http://ged.msu.edu/papers/2012-diginorm) to discard redundant data and reduce sampling variation and sequencing errors. The normalized dataset was assembled using Trinity^24^, SOAPdenovo and SOAPdenovo-Trans (http://soap.genomics.org.cn/) into contiguous sequences (contigs). Reads before assembly were re-mapped to contigs and the result was used to calculate number of reads for each contig. The use of several assembly algorithms and re-mapping of all singleton reads to assembled contigs were used to validate assembly results^19^,^25^. Contigs that were assembled by at least one of the assemblers were retained. Overall, 76322 contigs were assembled. For 65443 of them (86%) there were at least 4 raw reads (2 pair-end reads) that were remapped to the contig and the contig was then considered to be calid. Assembled, valid contigs were taxonomically classified by comparison against GenBank nucleotide database using Paracel (www.strikingdevelopment.com) blastn. To identify possible artifactual chimeras (contigs containing sequences originating from different DNA sequences) the sequence that aligned to its most closely related sequence in GenBank was divided into three equal segments. If at least one of the segments differed in similarity to the corresponding overlapping parts with more than 5% (for example, if segment 1 was 88% similar and segment 2 was 94% similar) the sequence was considered a possible chimera.

## Additional Information

**How to cite this article**: Smelov, V. *et al.* Detection of DNA viruses in prostate cancer. *Sci. Rep.* **6**, 25235; doi: 10.1038/srep25235 (2016).

## Figures and Tables

**Table 1:** Viruses found in prostate cancer cases and controls.

Virus group	Cases	Controls	Cases	Controls	OR (95%CI)
	Total reads	Total reads	Positive*	Positive	
Alphapapillomavirus 3 (HPV89)	4478	2	1	0	–
Alphapapillomavirus 8 (HPV40 & 43)	218	42	1	1	1 (0.06–17.9)
**Alphapapillomavirus**	**4696**	**44**	**2**	**1**	2.2 (0.17–27.6)
***Papillomaviridae***	***4696***	***44***	***2***	***1***	2.2 (0.17–27.6)
Choristoneura biennis entomopoxvirus L	6	0	1	0	–
Choristoneura rosaceana entomopoxvirus L	6	0	1	0	–
**Betaentomopoxvirus**	**12**	**2**	**1**	**0**	–
***Poxviridae***	***12***	***2***	***1***	***0***	–
Epstein-Barr virus	0	112	0	2	–
Cytomegalovirus	4	0	1	0	
***Herpesviridae***	**4**	**112**	**0**	**2**	–
JC polyomavirus	304	12	2	1	2.2 (0.17–27.6)
Merkel cell polyomavirus	7938	0	1	0	–
**Polyomavirus**	**8242**	**12**	**3**	**1**	3.6 (0.32–40.2)
***Polyomaviridae***	***8942***	***12***	***3***	***1***	3.6 (0.32–40.2)
**Total**	**13654**	**170**	**4**	**3**	1.5 (0.25–8.5)

^*^Considered positive at >4 reads per sample.

**Table 2:** Viruses in Prostatitis cases and controls.

Virus group	Cases	Controls	Cases	Controls	OR (95%CI)
	Total reads	Total reads	Positive*	Positive	
Choristoneura rosaceana entomopoxvirus L	2	38	0	2	–
**Betaentomopoxvirus**	**0**	**38**	**0**	**2**	–
***Poxviridae***	***2***	***38***	***0***	***2***	–
JC polyomavirus	0	14	0	1	–
**Polyomavirus**	**0**	**14**	**0**	**1**	–
***Polyomaviridae***	***0***	***14***	***0***	***1***	–
Megavirus chiliensis	14	4	2	0	–
**Mimiviridae_unclassified**	**14**	**4**	**2**	**0**	–
***Mimiviridae***	***14***	***4***	***2***	***0***	–
Phaeocystis globosa virus	0	32	0	1	–
**Prymnesiovirus**	**0**	**32**	**0**	**1**	–
***Phycodnaviridae***	***0***	***32***	***0***	***1***	–
IAS virus	0	46	0	1	–
**Unclassified**	**0**	**46**	**0**	**1**	–
***unclassified***	***0***	***46***	***0***	***1***	–
**Total**	**16**	**134**	**2**	**4**	0.25 (0.025–2.5)

^*^Considered positive at >4 reads per sample.
