# Corrigendum: Detection of DNA viruses in prostate cancer

**DOI:** 10.1038/srep26267

**Published:** 2016-05-31

**Authors:** Vitaly Smelov, Davit Bzhalava, Laila Sara Arroyo Mühr, Carina Eklund, Boris Komyakov, Andrey Gorelov, Joakim Dillner, Emilie Hultin

Scientific Reports 6: Article number: 2523510.1038/srep25235; published online: 04282016; updated: 05312016

In the original version of this Article, Affiliations 1 and 2 were not listed in the correct order. The correct affiliations are listed below:

Affiliation 1

Karolinska Institutet, Department of Laboratory Medicine, Stockholm, 141 86, Sweden.

Affiliation 2

International Agency for Research on Cancer, World Health Organization, Screening Group, Lyon, 69372, France.

This error has now been corrected in the PDF and HTML versions of the Article.

